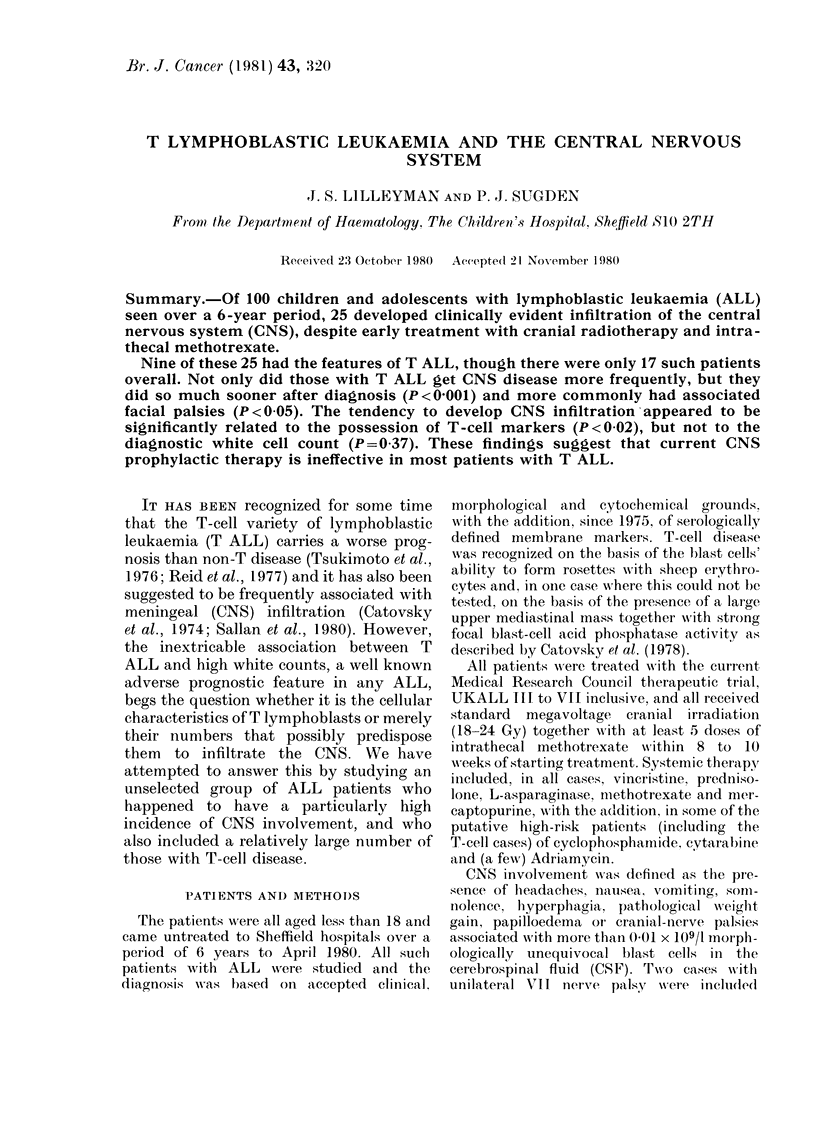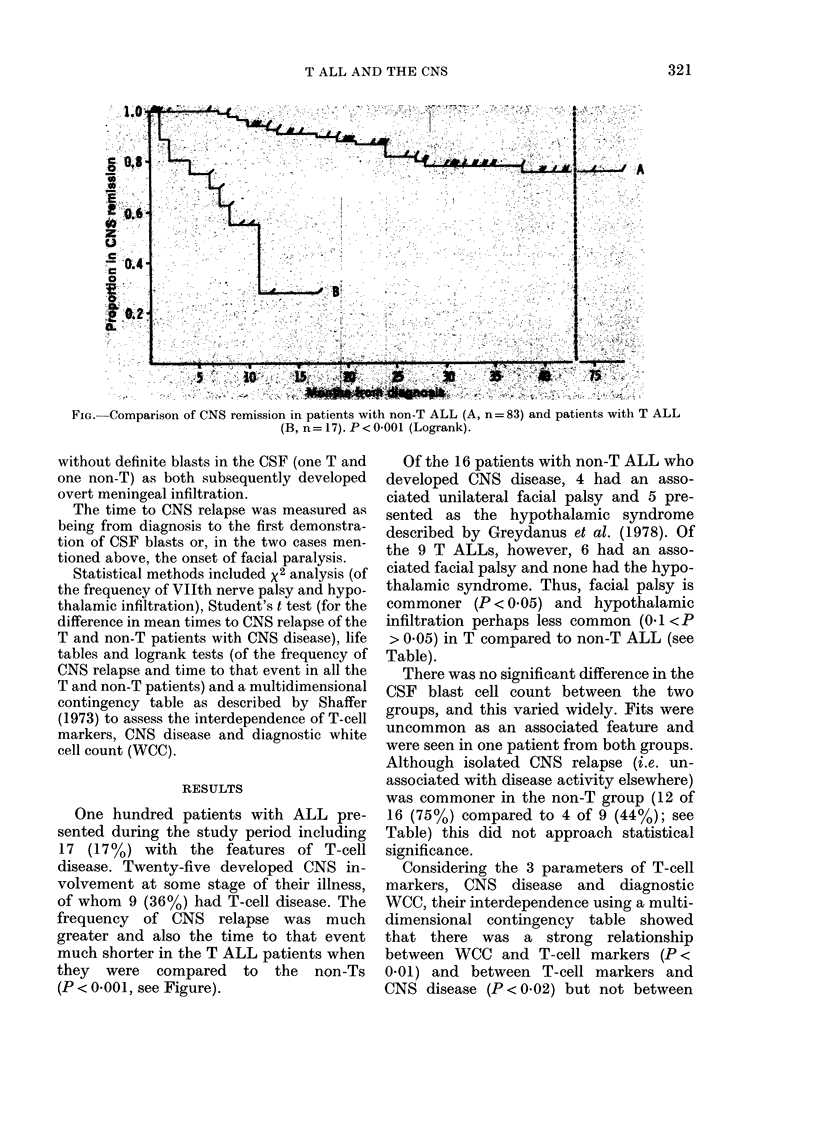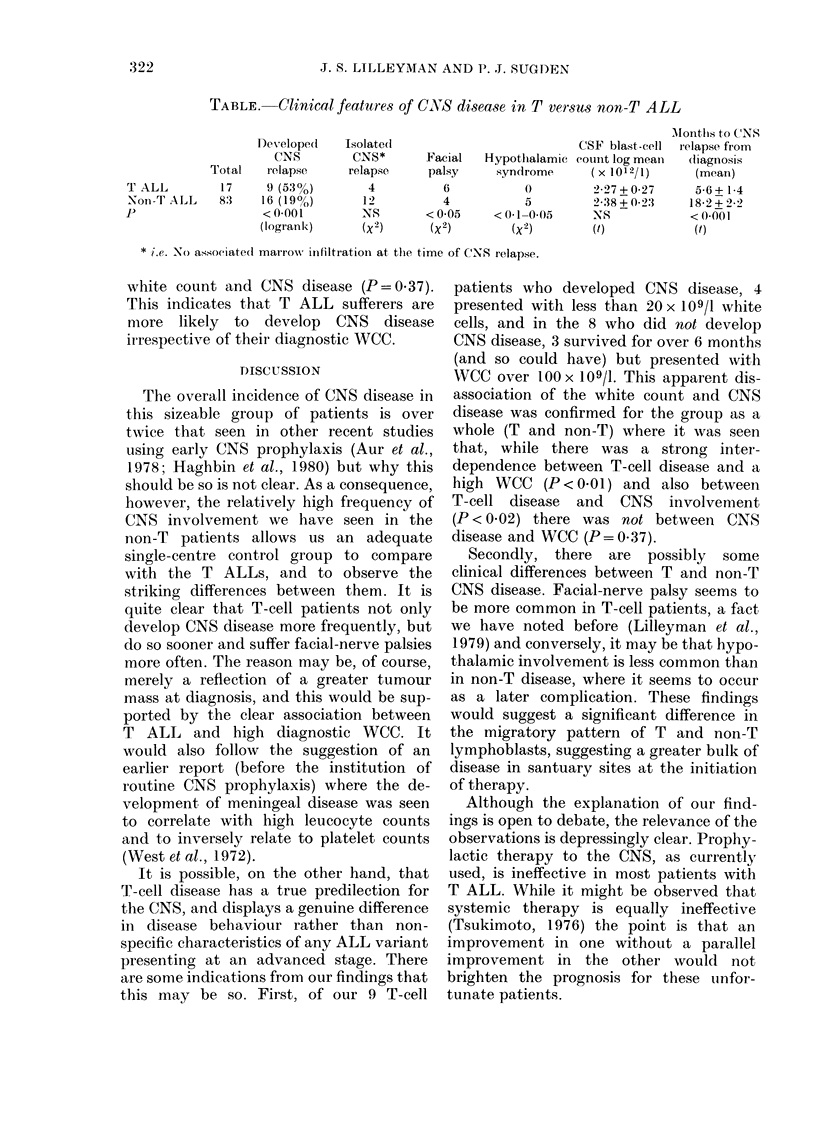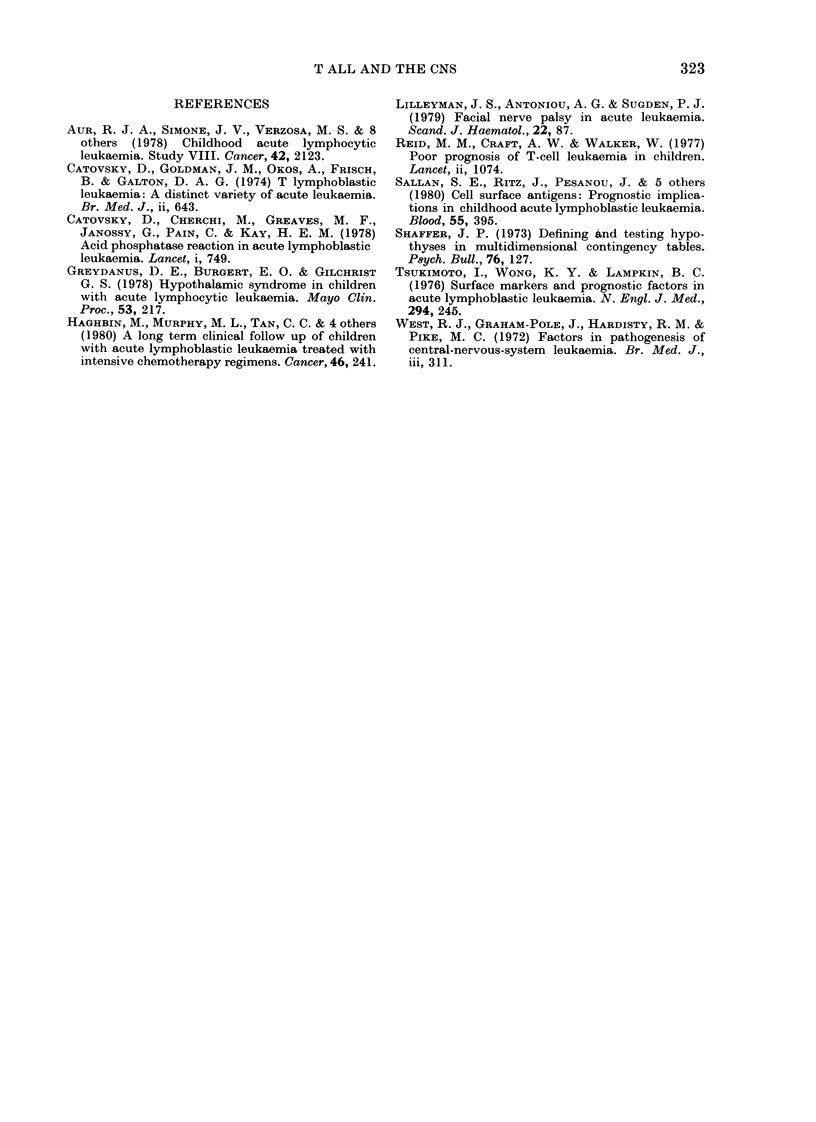# T lymphoblastic leukaemia and the central nervous system.

**DOI:** 10.1038/bjc.1981.50

**Published:** 1981-03

**Authors:** J. S. Lilleyman, P. J. Sugden

## Abstract

Of 100 children and adolescents with lymphoblastic leukaemia (ALL) seen over a 6-year period, 25 developed clinically evident infiltration of the central nervous system (CNS), despite early treatment with cranial radiotherapy and intrathecal methotrexate. Nine of these 25 had the features of T ALL, though there were only 17 such patients overall. Not only did those with T ALL get CNS disease more frequently, but they did so much sooner after diagnosis (P less than 0.001) and more commonly had associated facial palsies (P less than 0.05). The tendency to develop CNS infiltration appeared to be significantly related to the possession of T-cell markers (P less than 0.02), but not to the diagnostic white cell count (P = 0.37). These findings suggest that current CNS prophylactic therapy is ineffective in most patients with T ALL.


*Br. J. Cancer* (1981) **43**, 320

### T LYMPHOBLASTIC LEUKAEMIA AND THE CENTRAL NERVOUS SYSTEM

J. S. LILLEYMAN AND P. J. SUGDEN

*From the Department of Haematology, The Children's Hospital, Sheffield S10 2TH*

Received 23 October 1980 Accepted 21 November 1980

**Summary.—Of 100 children and adolescents with lymphoblastic leukaemia (ALL) seen over a 6-year period, 25 developed clinically evident infiltration of the central nervous system (CNS), despite early treatment with cranial radiotherapy and intrathecal methotrexate.**

**Nine of these 25 had the features of T ALL, though there were only 17 such patients overall. Not only did those with T ALL get CNS disease more frequently, but they did so much sooner after diagnosis ($P < 0{\cdot}001$) and more commonly had associated facial palsies ($P < 0{\cdot}05$). The tendency to develop CNS infiltration appeared to be significantly related to the possession of T-cell markers ($P < 0{\cdot}02$), but not to the diagnostic white cell count ($P = 0{\cdot}37$). These findings suggest that current CNS prophylactic therapy is ineffective in most patients with T ALL.**

It has been recognized for some time that the T-cell variety of lymphoblastic leukaemia (T ALL) carries a worse prognosis than non-T disease (Tsukimoto *et al.*, 1976; Reid *et al.*, 1977) and it has also been suggested to be frequently associated with meningeal (CNS) infiltration (Catovsky *et al.*, 1974; Sallan *et al.*, 1980). However, the inextricable association between T ALL and high white counts, a well known adverse prognostic feature in any ALL, begs the question whether it is the cellular characteristics of T lymphoblasts or merely their numbers that possibly predispose them to infiltrate the CNS. We have attempted to answer this by studying an unselected group of ALL patients who happened to have a particularly high incidence of CNS involvement, and who also included a relatively large number of those with T-cell disease.

### PATIENTS AND METHODS

The patients were all aged less than 18 and came untreated to Sheffield hospitals over a period of 6 years to April 1980. All such patients with ALL were studied and the diagnosis was based on accepted clinical, morphological and cytochemical grounds, with the addition, since 1975, of serologically defined membrane markers. T-cell disease was recognized on the basis of the blast cells' ability to form rosettes with sheep erythrocytes and, in one case where this could not be tested, on the basis of the presence of a large upper mediastinal mass together with strong focal blast-cell acid phosphatase activity as described by Catovsky *et al.* (1978).

All patients were treated with the current Medical Research Council therapeutic trial, UKALL III to VII inclusive, and all received standard megavoltage cranial irradiation (18–24 Gy) together with at least 5 doses of intrathecal methotrexate within 8 to 10 weeks of starting treatment. Systemic therapy included, in all cases, vincristine, prednisolone, L-asparaginase, methotrexate and mercaptopurine, with the addition, in some of the putative high-risk patients (including the T-cell cases) of cyclophosphamide, cytarabine and (a few) Adriamycin.

CNS involvement was defined as the presence of headaches, nausea, vomiting, somnolence, hyperphagia, pathological weight gain, papilloedema or cranial-nerve palsies associated with more than $0{\cdot}01 \times 10^9$/l morphologically unequivocal blast cells in the cerebrospinal fluid (CSF). Two cases with unilateral VII nerve palsy were included

FIG.—Comparison of CNS remission in patients with non-T ALL (A, n = 83) and patients with T ALL (B, n = 17). $P < 0{\cdot}001$ (Logrank).

without definite blasts in the CSF (one T and one non-T) as both subsequently developed overt meningeal infiltration.

The time to CNS relapse was measured as being from diagnosis to the first demonstration of CSF blasts or, in the two cases mentioned above, the onset of facial paralysis.

Statistical methods included $\chi^2$ analysis (of the frequency of VIIth nerve palsy and hypothalamic infiltration), Student's *t* test (for the difference in mean times to CNS relapse of the T and non-T patients with CNS disease), life tables and logrank tests (of the frequency of CNS relapse and time to that event in all the T and non-T patients) and a multidimensional contingency table as described by Shaffer (1973) to assess the interdependence of T-cell markers, CNS disease and diagnostic white cell count (WCC).

### RESULTS

One hundred patients with ALL presented during the study period including 17 (17%) with the features of T-cell disease. Twenty-five developed CNS involvement at some stage of their illness, of whom 9 (36%) had T-cell disease. The frequency of CNS relapse was much greater and also the time to that event much shorter in the T ALL patients when they were compared to the non-Ts ($P < 0{\cdot}001$, see Figure).

Of the 16 patients with non-T ALL who developed CNS disease, 4 had an associated unilateral facial palsy and 5 presented as the hypothalamic syndrome described by Greydanus *et al.* (1978). Of the 9 T ALLs, however, 6 had an associated facial palsy and none had the hypothalamic syndrome. Thus, facial palsy is commoner ($P < 0{\cdot}05$) and hypothalamic infiltration perhaps less common ($0{\cdot}1 < P > 0{\cdot}05$) in T compared to non-T ALL (see Table).

There was no significant difference in the CSF blast cell count between the two groups, and this varied widely. Fits were uncommon as an associated feature and were seen in one patient from both groups. Although isolated CNS relapse (*i.e.* unassociated with disease activity elsewhere) was commoner in the non-T group (12 of 16 (75%) compared to 4 of 9 (44%); see Table) this did not approach statistical significance.

Considering the 3 parameters of T-cell markers, CNS disease and diagnostic WCC, their interdependence using a multidimensional contingency table showed that there was a strong relationship between WCC and T-cell markers ($P < 0{\cdot}01$) and between T-cell markers and CNS disease ($P < 0{\cdot}02$) but not between

TABLE.—*Clinical features of CNS disease in T versus non-T ALL*

| | Total | Developed CNS relapse | Isolated CNS* relapse | Facial palsy | Hypothalamic syndrome | CSF blast-cell count log mean ($\times 10^{12}$/l) | Months to CNS relapse from diagnosis (mean) |
|---|---|---|---|---|---|---|---|
| T ALL | 17 | 9 (53%) | 4 | 6 | 0 | $2 \cdot 27 \pm 0 \cdot 27$ | $5 \cdot 6 \pm 1 \cdot 4$ |
| Non-T ALL | 83 | 16 (19%) | 12 | 4 | 5 | $2 \cdot 38 \pm 0 \cdot 23$ | $18 \cdot 2 \pm 2 \cdot 2$ |
| $P$ | | $< 0 \cdot 001$ (logrank) | NS ($\chi^2$) | $< 0 \cdot 05$ ($\chi^2$) | $< 0 \cdot 1$–$0 \cdot 05$ ($\chi^2$) | NS ($t$) | $< 0 \cdot 001$ ($t$) |

* *i.e.* No associated marrow infiltration at the time of CNS relapse.

white count and CNS disease ($P = 0 \cdot 37$). This indicates that T ALL sufferers are more likely to develop CNS disease irrespective of their diagnostic WCC.

### DISCUSSION

The overall incidence of CNS disease in this sizeable group of patients is over twice that seen in other recent studies using early CNS prophylaxis (Aur *et al.*, 1978; Haghbin *et al.*, 1980) but why this should be so is not clear. As a consequence, however, the relatively high frequency of CNS involvement we have seen in the non-T patients allows us an adequate single-centre control group to compare with the T ALLs, and to observe the striking differences between them. It is quite clear that T-cell patients not only develop CNS disease more frequently, but do so sooner and suffer facial-nerve palsies more often. The reason may be, of course, merely a reflection of a greater tumour mass at diagnosis, and this would be supported by the clear association between T ALL and high diagnostic WCC. It would also follow the suggestion of an earlier report (before the institution of routine CNS prophylaxis) where the development of meningeal disease was seen to correlate with high leucocyte counts and to inversely relate to platelet counts (West *et al.*, 1972).

It is possible, on the other hand, that T-cell disease has a true predilection for the CNS, and displays a genuine difference in disease behaviour rather than non-specific characteristics of any ALL variant presenting at an advanced stage. There are some indications from our findings that this may be so. First, of our 9 T-cell patients who developed CNS disease, 4 presented with less than $20 \times 10^9$/l white cells, and in the 8 who did *not* develop CNS disease, 3 survived for over 6 months (and so could have) but presented with WCC over $100 \times 10^9$/l. This apparent dissociation of the white count and CNS disease was confirmed for the group as a whole (T and non-T) where it was seen that, while there was a strong interdependence between T-cell disease and a high WCC ($P < 0 \cdot 01$) and also between T-cell disease and CNS involvement ($P < 0 \cdot 02$) there was *not* between CNS disease and WCC ($P = 0 \cdot 37$).

Secondly, there are possibly some clinical differences between T and non-T CNS disease. Facial-nerve palsy seems to be more common in T-cell patients, a fact we have noted before (Lilleyman *et al.*, 1979) and conversely, it may be that hypothalamic involvement is less common than in non-T disease, where it seems to occur as a later complication. These findings would suggest a significant difference in the migratory pattern of T and non-T lymphoblasts, suggesting a greater bulk of disease in santuary sites at the initiation of therapy.

Although the explanation of our findings is open to debate, the relevance of the observations is depressingly clear. Prophylactic therapy to the CNS, as currently used, is ineffective in most patients with T ALL. While it might be observed that systemic therapy is equally ineffective (Tsukimoto, 1976) the point is that an improvement in one without a parallel improvement in the other would not brighten the prognosis for these unfortunate patients.

### REFERENCES

AUR, R. J. A., SIMONE, J. V., VERZOSA, M. S. & 8 others (1978) Childhood acute lymphocytic leukaemia. Study VIII. *Cancer*, **42**, 2123.

CATOVSKY, D., GOLDMAN, J. M., OKOS, A., FRISCH, B. & GALTON, D. A. G. (1974) T lymphoblastic leukaemia: A distinct variety of acute leukaemia. *Br. Med. J.*, ii, 643.

CATOVSKY, D., CHERCHI, M., GREAVES, M. F., JANOSSY, G., PAIN, C. & KAY, H. E. M. (1978) Acid phosphatase reaction in acute lymphoblastic leukaemia. *Lancet*, i, 749.

GREYDANUS, D. E., BURGERT, E. O. & GILCHRIST G. S. (1978) Hypothalamic syndrome in children with acute lymphocytic leukaemia. *Mayo Clin. Proc.*, **53**, 217.

HAGHBIN, M., MURPHY, M. L., TAN, C. C. & 4 others (1980) A long term clinical follow up of children with acute lymphoblastic leukaemia treated with intensive chemotherapy regimens. *Cancer*, **46**, 241.

LILLEYMAN, J. S., ANTONIOU, A. G. & SUGDEN, P. J. (1979) Facial nerve palsy in acute leukaemia. *Scand. J. Haematol.*, **22**, 87.

REID, M. M., CRAFT, A. W. & WALKER, W. (1977) Poor prognosis of T-cell leukaemia in children. *Lancet*, ii, 1074.

SALLAN, S. E., RITZ, J., PESANOU, J. & 5 others (1980) Cell surface antigens: Prognostic implications in childhood acute lymphoblastic leukaemia. *Blood*, **55**, 395.

SHAFFER, J. P. (1973) Defining and testing hypothyses in multidimensional contingency tables. *Psych. Bull.*, **76**, 127.

TSUKIMOTO, I., WONG, K. Y. & LAMPKIN, B. C. (1976) Surface markers and prognostic factors in acute lymphoblastic leukaemia. *N. Engl. J. Med.*, **294**, 245.

WEST, R. J., GRAHAM-POLE, J., HARDISTY, R. M. & PIKE, M. C. (1972) Factors in pathogenesis of central-nervous-system leukaemia. *Br. Med. J.*, iii, 311.